# A Metataxonomic Approach Could Be Considered for Cattle Clinical Mastitis Diagnostics

**DOI:** 10.3389/fvets.2017.00036

**Published:** 2017-03-10

**Authors:** Joanne W. H. Oultram, Erika K. Ganda, Sarah C. Boulding, Rodrigo C. Bicalho, Georgios Oikonomou

**Affiliations:** ^1^Department of Livestock Health and Welfare, Institute of Veterinary Science, University of Liverpool, Neston, UK; ^2^Department of Population Medicine and Diagnostic Sciences, College of Veterinary Medicine, Cornell University, Ithaca, NY, USA; ^3^Department of Epidemiology and Population Health, Institute of Infection and Global Health, University of Liverpool, Neston, UK

**Keywords:** metataxonomics, mastitis, cattle, diagnostics, sequencing

## Abstract

Mastitis is one of the most costly diseases affecting the dairy industry, and identification of the causative microorganism(s) is essential. Here, we report the use of next-generation sequencing of bacterial 16S rRNA genes for clinical mastitis diagnosis. We used 65 paired milk samples, collected from the mastitic and a contralateral healthy quarter of mastitic dairy cattle to evaluate the technique as a potential alternative to bacterial culture or targeted PCR. One large commercial dairy farm was used, with one trained veterinarian collecting the milk samples. The 16S rRNA genes were individually amplified and sequenced using the MiSeq platform. The MiSeq Reporter was used in order to analyze the obtained sequences. Cattle were categorized according to whether or not 1 of the 10 most abundant bacterial genera in the mastitic quarter exhibited an increase in relative abundance between the healthy and mastitic quarters equal to, or exceeding, twofold. We suggest that this increase in relative abundance is indicative of the genus being a causative mastitis pathogen. Well-known mastitis-causing pathogens such as *Streptococcus uberis* and *Staphylococcus* spp. were identified in most cattle. We were able to diagnose 53 out of the 65 studied cases and identify potential new mastitis pathogens such as *Sneathia sanguinegens* and *Listeria innocua*, which are difficult to identify by bacterial culture because of their fastidious nature.

## Introduction

Mastitis is one of the most important diseases in dairy herds worldwide, compromising animal welfare and causing considerable economic loses (1–3). As bacterial resistance to antibiotics and the demand for milk increase, the need for efficient mastitis diagnostics is becoming ever more evident (4). Rapid identification of the causative microorganisms of mastitis permits prompt treatment and reduction in antibiotic use (5, 6) by reducing total duration of treatment and the unnecessary use of broad spectrum antimicrobials. The gold standard for identification of the causative pathogen is by bacterial culture, which uses standards set by the National Mastitis Council. Culture, however, has an inherent bias toward organisms that are able grow on the selected media. Up to 40% of milk samples collected from cows with clinical mastitis will yield negative results by aerobic culture (7).

An increase in the use of the culture-independent alternatives to identify bacterial DNA in milk samples has overcome some of the limitations of bacterial culture being rapid (results in 1–2 days), unaffected by antibiotic administration pre-sampling and having increased the sensitivity of detection of known mastitis-causing organisms, as well as enabling the investigation of potential new pathogens. Advances in next-generation sequencing allow the in-depth investigation of clinical samples’ microbiomes and determining their taxonomic composition including unculturable species (8). Shotgun sequencing is still prohibitively expensive in a commercial clinical setting, whereas a metataxonomic (16S rRNA gene sequencing) approach could be a relatively rapid and cost-effective method for assessing bacterial diversity and abundance (9, 10).

Our group has previously used metataxonomics and described the microbial diversity in bovine mastitic and healthy milk; this was a cross-sectional study of 136 samples of mastitic milk and 20 samples of uninfected milk as defined by having a low cell count. Results were compared to results obtained by culturing (9). The mastitis pathogens identified by culture were generally among the most frequent organisms detected by sequencing, and in some cases (*Escherichia coli, Klebsiella* spp., and *Streptococcus uberis* mastitis), the single most prevalent microorganism. In samples that were aerobic culture negative, pyrosequencing identified DNA of bacteria that are known to cause mastitis, DNA of bacteria that are known pathogens but have so far not been associated with mastitis, and DNA of bacteria that are currently not known to be pathogens.

The use of the Illumina MiSec sequencing platform and the MiSeq Reporter for sequences analysis could further decrease the cost of metataxonomic studies facilitating at the same time a speedier analysis of the obtained sequences. Here, we use a metataxonomic approach in order to identify potential clinical mastitis pathogens and further evaluate its potential uses as a clinical diagnostic tool.

## Materials and Methods

### Ethics Statement

The research protocol was reviewed and approved by the Cornell University Institutional Animal Care and Use Committee (protocol number 2013-0056). The methods were carried out in accordance with the approved guidelines.

### Animals, Facilities, and Sample Collection

The study was conducted using cows from a commercial dairy herd near Ithaca, NY, USA, milking approximately 2,800 cows. Primiparous and multiparous cows were housed separately in free-stall barns, the concrete stalls being bedded using mattresses and manure solids. Cows were fed a total mixed ration to meet or exceed the nutrient requirements of a 650 kg lactating Holstein cow producing 45 kg/day of milk containing 3.5% fat and 3.2% protein and assuming a dry matter intake of 25 kg/day (11). Cows were milked three times daily in a double 52 milking parlor.

Cows with clinical mastitis were identified using the parlor computer system, which identified those with a significant reduction in milk production; these animals were further examined, and if visual assessment of milk revealed flakes, clots, or serous milk, a sample for on-farm culture was taken by trained farm personnel and the animal moved to the hospital pen. Additionally, cows identified as having abnormal milk during routine fore stripping in the milking parlor were similarly sampled and moved to the hospital pen.

Milk samples for metataxonomic analysis were collected aseptically by a trained veterinarian, following the recommendations of the National Mastitis Council mastitis handbook, during the morning milking the day after the cows entered the hospital pen. Teat ends were cleaned with routine pre-dipping technique and disinfected with 70% ethanol, and the first streams of milk were discarded. Sixty-five cows were sampled, 10-ml milk being extracted from both the mastitic quarter and a contralateral non-mastitic quarter. The samples were transported on ice for DNA extraction.

### DNA Extraction

DNA was extracted from each collected sample separately. Also, 10 ml of milk was centrifuged at 4°C and 9,000 rpm for 30 min. The fat and majority of supernatant were removed by suction and 300 μl supernatant retained to resuspend the pellet. The milk pellet and the remaining supernatant were vortexed and transferred to a sterile micro centrifuge tube using a sterile transfer pipette, before being incubated at 40°C for 12 h with 180 μl of tissue lysis buffer ATL (Qiagen, Valencia, CA, USA), 40 μl of proteinase K (IBI Scientific), and 20 μl of lysozyme solution (10 mg/ml) to maximize bacterial DNA extraction.

Isolation of genomic DNA was performed on 250 μl of post-incubation mixture pipetted into PowerBead Tubes (PowerSoil^®^ DNA Isolation kit, MO BIO Laboratories, Inc., Carlsbad, CA, USA) and settled in a Mini-Beadbeater-8 (Biospec Products, Battersville, OK, USA) for microbial cell disruption. DNA extraction was performed using a PowerSoil DNA Isolation Kit (MO BIO Laboratory Inc.) following the manufacturer’s recommendation. DNA concentration and purity were evaluated by optical density using a NanoDrop ND-1000 spectrophotometer (NanoDrop Technologies, Rockland, DE, USA) at wavelengths of 230, 260, and 280 nm.

### PCR Amplification of the V4 Hypervariable Region of Bacterial 16S rRNA Genes

For amplification of the V4 hypervariable region of the bacterial 16S rRNA gene, primers 515F and 806R were used according to a previously described method (12) optimized for the Illumina MiSeq platform. The Earth Microbiome Project (13) was used to select 140 different 12-bp error-correcting Golay barcodes for the 16S rRNA PCR, as previously described (12). The 5′-barcoded amplicons were generated in triplicate using 12–300 ng DNA template, 1× GoTaq Green Master Mix (Promega, Madison, WI, USA), and 10 μM of each primer. The PCR conditions for the 16S rRNA gene consisted of an initial denaturing step of 94°C for 3 min, followed by 35 cycles of 94°C for 45 s, 50°C for 1 min, and 72°C for 90 s, and a final elongation step of 72°C for 10 min. Replicate amplicons were pooled and purified with a Gel PCR DNA Fragment Extraction kit (IBI Scientific) and visualized by electrophoresis through 1.2% (wt/vol) agarose gels stained with 0.5 mg/ml ethidium bromide before sequencing. Blank controls, in which no DNA was added to the reaction, were performed. Purified amplicon DNA was quantified using the Qubit Flurometer (Life Technologies Corporation, Carlsbad, CA, USA).

### Sequence Library

Amplicon aliquots were standardized to the same concentration and then pooled. Final equimolar libraries were sequenced using the MiSeq reagent kit V2 for 300 cycles on the MiSeq platform (Illumina, Inc., San Diego, CA, USA). Gene sequences were processed using the 16S Metagenomics workflow in the MiSeq Reporter analysis software version 2.5 based on quality scores generated by real-time analysis during the sequencing run. Quality-filtered indexed reads were demultiplexed for generation of individual FASTQ files and aligned using the banded Smith–Waterman method of the Illumina-curated version of the Greengenes database for taxonomic classification of milk microbes. The output of this workflow was a classification of reads at multiple taxonomic levels: kingdom, phylum, class, order, family, genus, and species. To calculate relative abundance, we divided the number of sequences belonging to a specific species by the total number of sequences obtained from the specific sample. The same was done with information obtained at the bacterial genus (instead of species) level.

### Data Analysis

The 10 most abundant bacterial species in each mastitic quarter were identified. The increase in relative abundance of these bacteria in the mastitic quarter, comparing to the healthy one was calculated (dividing the relative abundance in the mastitis quarter by the relative abundance in the healthy one). A minimum twofold increase in relative abundance was taken to indicate probable pathogenicity. Subsequently, the relative abundances in healthy and mastitic quarters of the bacteria identified as potential pathogens were compared with the use of the non-parametric Wilcoxon exact test. This was not done for putative pathogens that were only identified in one mastitis case.

## Results

**Figure 1:**
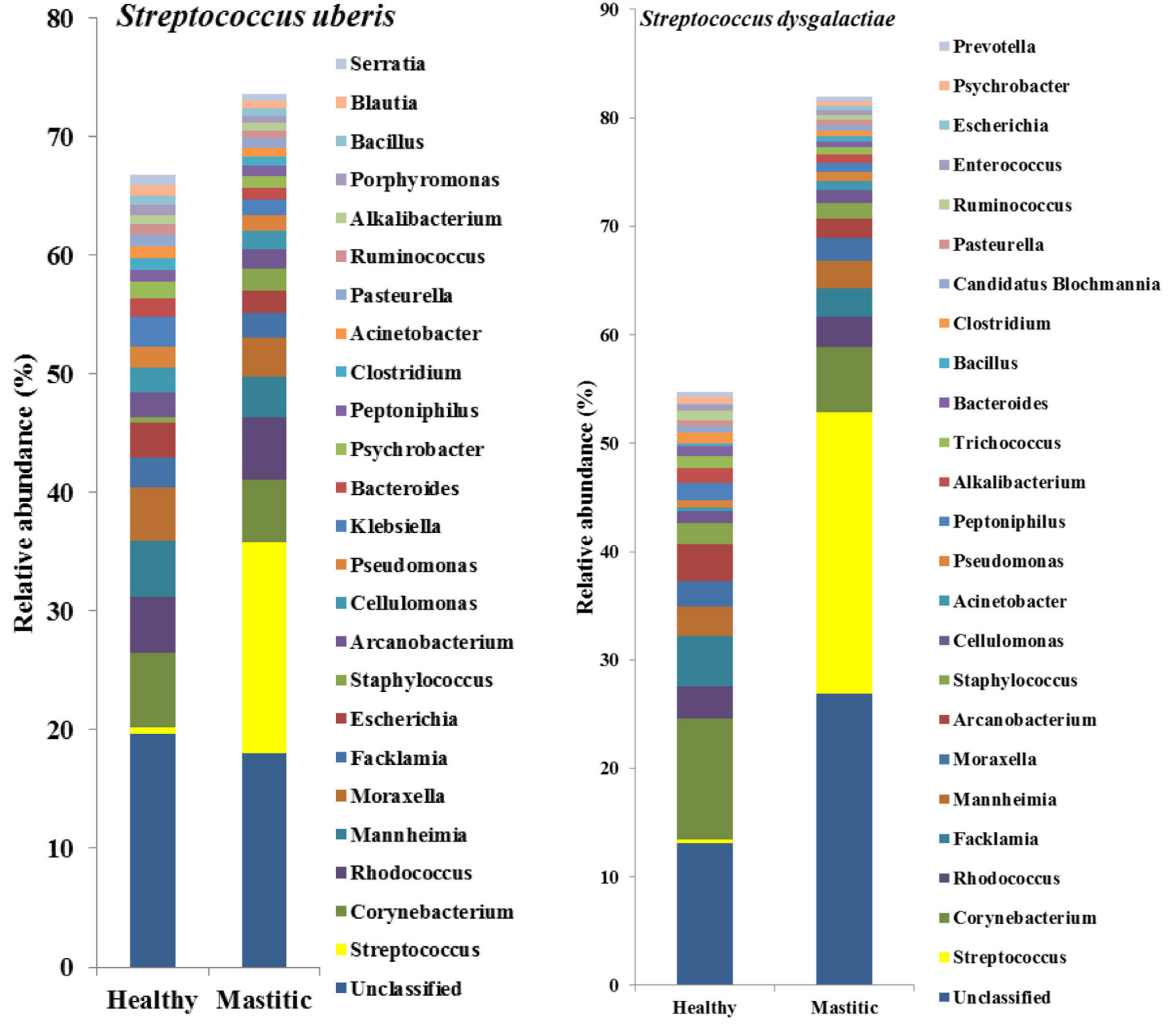

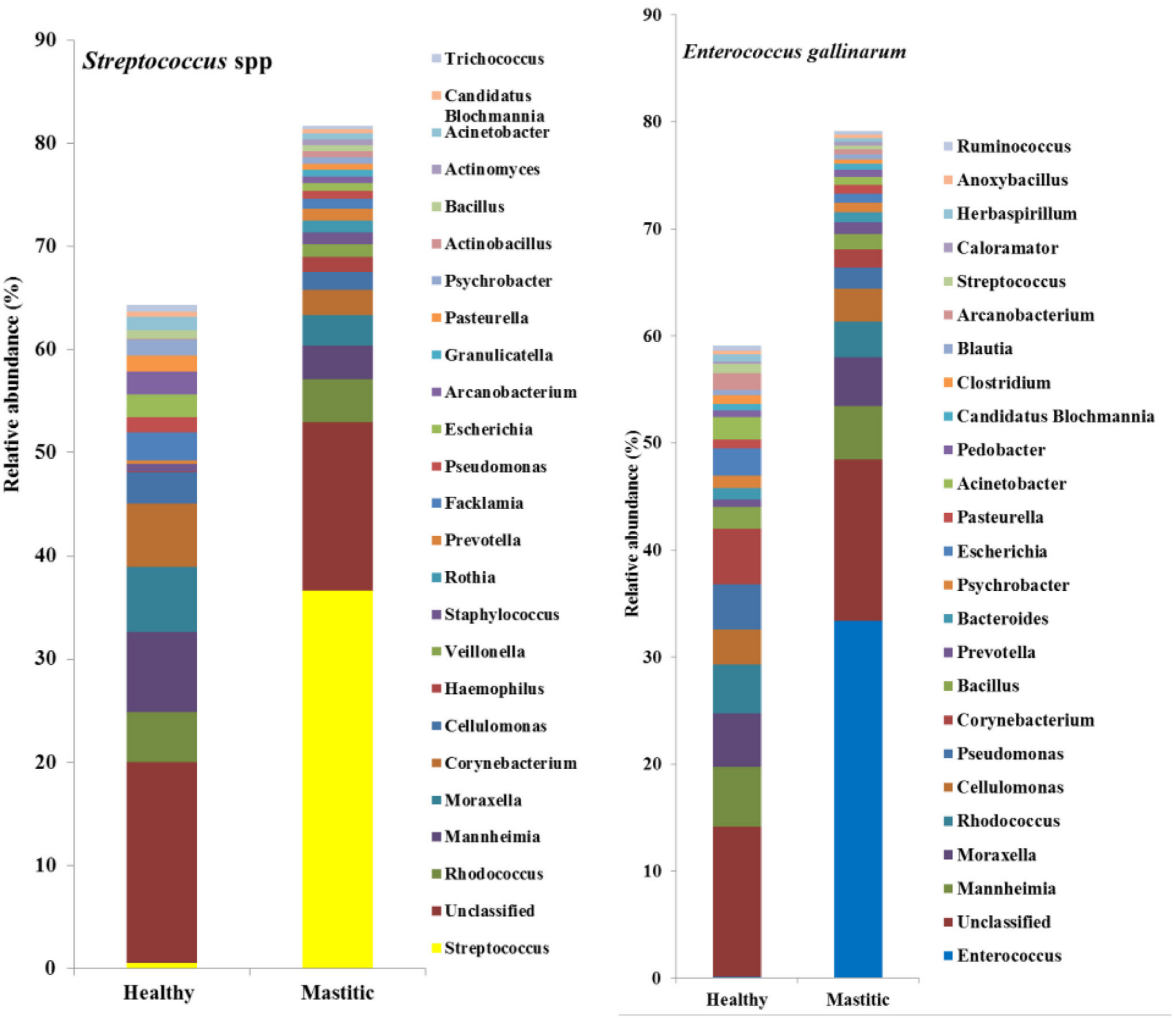
**Mean relative abundance of the 25 most prevalent genera in samples diagnosed as *Streptococcus uberis, Streptococcus dysgalactiae*, other *Streptococcus* spp., or *Enterococcus gallinarum***.

**Figure 2:**
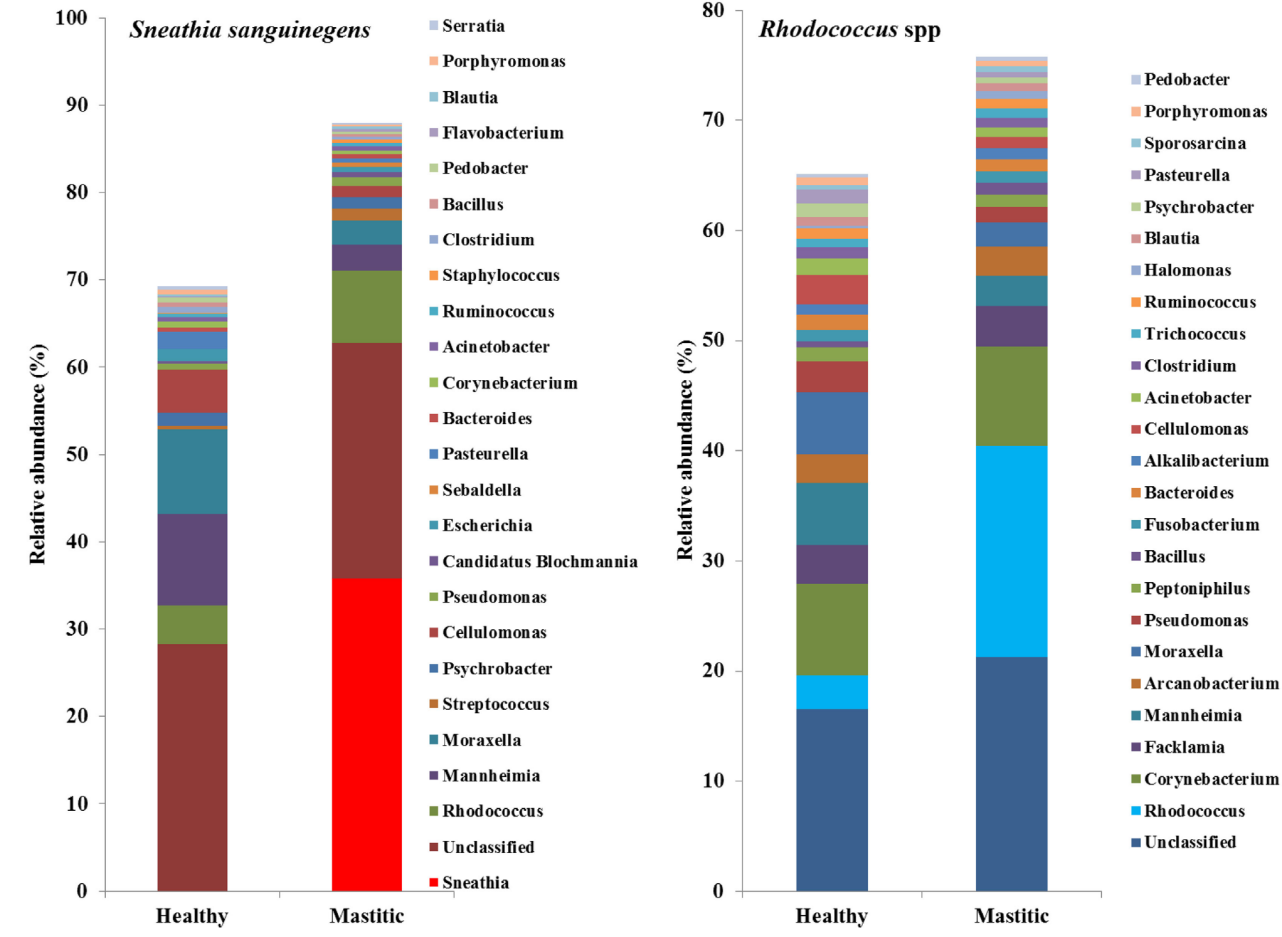

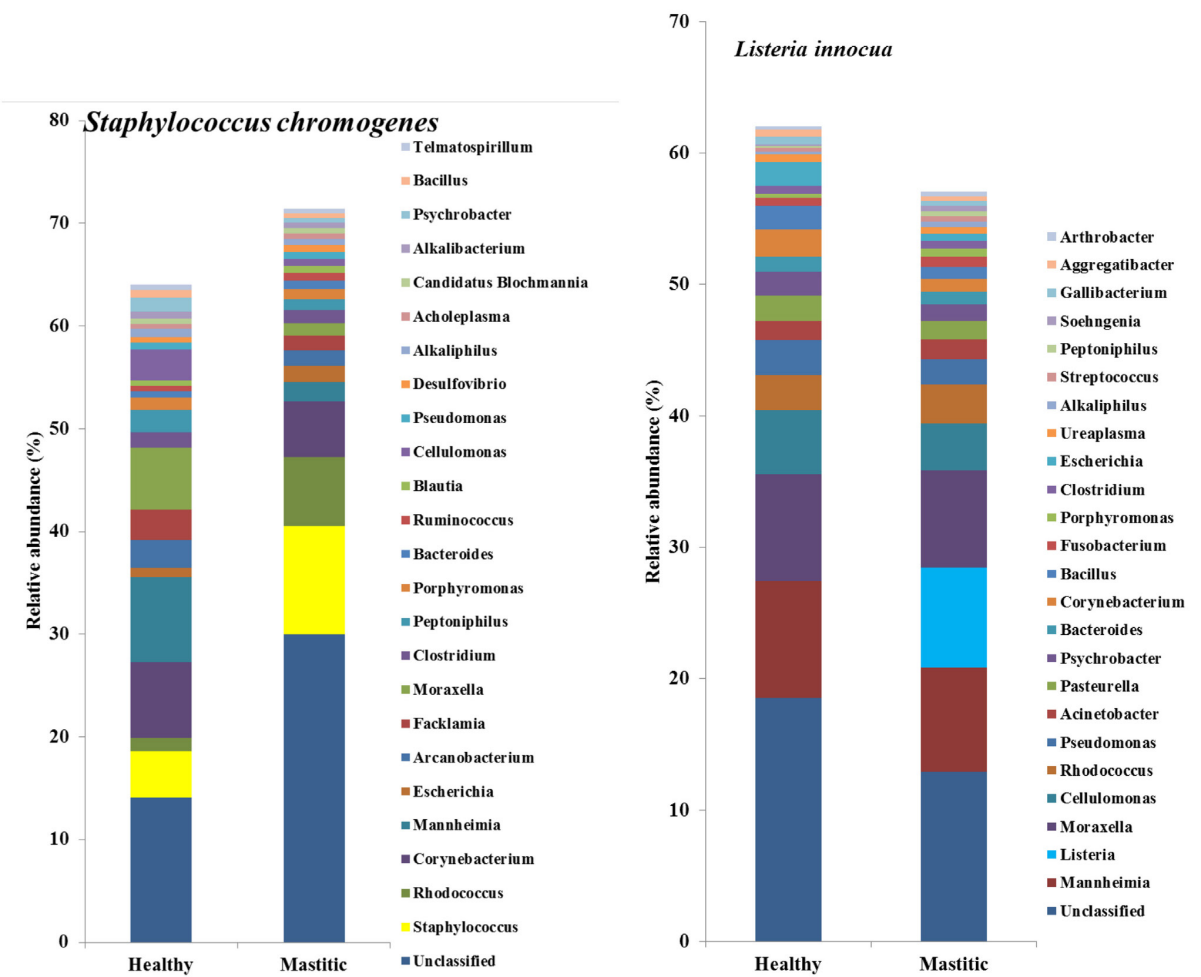
**Mean relative abundance of the 25 most prevalent genera in samples diagnosed as *Sneathia sanguinegens, Rhodococcus* spp., *Staphylococcus chromogenes*, or *Listeria innocua***.

In 53 of the 65 sampled cattle (81%), we were able to identify a bacterial species among the 10 most abundant in the mastitic quarter that had a relative abundance at least double that of itself in the healthy quarter. Results regarding these 53 cows are presented in Table 1. In the remaining 12 cows (19% of those sampled), the increase in bacterial abundance between the mastitic and healthy quarters was less than twofold. Mean relative abundance of the 25 most prevalent genera in samples diagnosed as *S. uberis, Streptococcus dysgalactiae*, other *Streptococcus* spp., or *Enterococcus gallinarum* is presented in Figure 1. Mean relative abundance of the 25 most prevalent genera in samples diagnosed as *Sneathia sanguinegens, Rhodococcus* spp., *Staphylococcus chromogenes*, or *Listeria innocua* is presented in Figure 2. Mean relative abundance of the 25 most prevalent genera in samples diagnosed as *Corynebacterium* spp., *Staphylococcus carnosus, E. coli*, and *Pasteurella dagmatis* is presented in Figure S1 in Supplementary Material. Mean relative abundance of the 25 most prevalent genera in samples diagnosed as *Moraxella lacumata, Faclamia hominis, Peptoniphilus methioninivorax*, and *Pseudomonas azotoformans* is presented in Figure S2 in Supplementary Material.

**Table 1:** **Mean relative abundance in healthy and mastitic quarters (percent ± SE of the mean) of bacterial species identified as the potential mastitis causative agents**.

Species	*N*	Healthy quarter	Mastitic quarter	*P*-value
*Streptococcus uberis*	23	0.23 ± 0.09	31.93 ± 5.81	<0.0001
*Streptococcus dysgalactiae*	4	0.011 ± 0.0016	17.39 ± 8.56	0.01
*Streptococcus* spp.	3	0.003 ± 0.003	2.10 ± 0.55	0.049
*Staphylococcus chromogenes*	2	0.01 ± 0.003	9.03 ± 7.73	0.17
*Corynebacterium* spp.	3	4.96 ± 3.01	11.35 ± 3.62	0.10
*Enterococcus gallinarum*	2	0.01 ± 0.003	12.64 ± 6.72	0.16
*Listeria innocua*	2	0.01 ± 0.006	7.60 ± 4.12	0.16
*Rhodococcus* spp.	4	1.01 ± 0.37	4.83 ± 1.69	0.01
*Sneathia sanguinegens*	2	0.06 ± 0.03	35.77 ± 32.74	0.16
*Escherichia coli*	1	0.11	13.91	
*Moraxella lacumata*	1	0.25	2.92	
*Staphylococcus carnosus*	1	0.003	1.73	
*Pasteurella dagmatis*	1	0.02	7.17	
*Acholeplasma ales*	1	0.34	1.32	
*Faclamia hominis*	1	2.65	8.05	
*Peptoniphilus methioninivorax*	1	1.1	2.29	
*Pseudomonas azotoformans*	1	0.33	2.65	

*Presented P values were obtained with the use of the Wilcoxon exact test. For species identified as potential causative agents in only one cow, the actual relative abundances are presented; P values were not obtained*.

*N, number of cows for which the indicated species was identified as the major pathogen*.

The most prevalent bacterial genus was *Streptococcus* spp., which was identified as the potential causative microorganism in 30 of the 53 mastitic quarter cases. These bacterial genuses comprised 23 *S. uberis*, 4 *S. dysgalactiae* (which exhibited the highest individual bacterial increase in relative abundance, a 3,916-fold increase in one cow), and 3 other *Streptococcus* spp. The second most abundant genus was *Staphylococcus* spp., and more specifically, the coagulase negative staphylococci (CNS) *S. carnosus* in one cow and *S. chromogenes* in two cows. *S. sanguinegens* and *Rhodococcus* spp. were identified as the potential pathogens in the mastitic quarters of two and four cattle, respectively. *Corynebacterium* spp. were identified as the potential pathogens in three cases, while *E. gallinarum* was implicated in two cases.

*Escherichia coli, M. lacumata, P. dagmatis, Acholeplasma ales, F. hominis, P. azotoformans*, and *P. methioninivorax* were also identified as being the bacterium exhibiting the greatest increase in relative abundance in single cows. However, when the sample diagnosed as *F. hominis* was analyzed at the genus level (Figure S2 in Supplementary Material), it was revealed that this was probably a *Streptococcus* spp. mastitis case that was misdiagnosed at the species level analysis. Additionally, the genus level analysis for the two samples diagnosed as *P. azotoformans* and *P. methioninivorax* (Figure S2 in Supplementary Material) is not as convincing of the validity of this diagnosis as it is in most of the other cases, and the possibility of a different unidentified (potentially non-bacterial) causative agent should not be excluded.

## Discussion

If it is accepted that an increase in bacterial sequences abundance between a healthy quarter and one which is mastitic indicates pathogenicity, then most of the cows in our study exhibited increases such that the case of mastitis could be attributed to specific bacteria. We used a metataxonomic approach not in order to conduct a study on the bovine milk microbiome in health and disease as we and other research groups have done previously (9, 14, 15), but in order to evaluate its potential use in mastitis diagnostics. In most of our samples, some well-recognized mastitis pathogens were described. Additionally, other bacteria, not yet recognized as mastitis pathogens, were identified at significant abundances in quarters in which no other known pathogen was identified.

Admittedly, more research is warranted before our approach is considered as an alternative for cattle mastitis diagnostics. Additionally, certain limitations do have to be considered here. Using a 16S rRNA approach, we were only able to describe bacterial populations. Any yeast- or fungus-related mastitis would not be detected. There is also the chance that such a mastitis pathogen would have caused a disturbance to the mastitic quarter microbiome leading to differences between the mastitic and the healthy quarter and potential false positives. Inclusion of 18S rRNA sequencing can in the future alleviate this problem. Viral mastitis is also not considered here, but this is a common problem for all the diagnostic methods currently employed for every day bovine mastitis diagnostics.

The most commonly identified bacterium here was *S. uberis*, a pathogen of environmental origin (16), which also exhibits cow to cow transmission (16, 17). United States studies have shown that the most prevalent pathogens causing clinical mastitis are environmental in origin (6, 18–20), and the use of manure solids as substrate in the herd’s stalls, which is also suggested to increase the prevalence of *S. uberis* (21), makes it unsurprising that *S. uberis* was identified at high prevalence in mastitic quarters in the study herd and lends validity to the use of DNA sequencing in the identification of mastitis pathogens. Similarly, *S. dysgalactiae*, which is associated with both environmental and contagious mastitis (22), and other *Streptococcus* species, which have previously been identified on teat skin and in milk including *Streptococcus bovis* and *Streptococcus canis* (23, 24), were listed among the 10 most prevalent bacteria in the study population.

Both CNS and coagulase positive *staphylococci* (CPS) were identified in the study samples. CPS (other than *Staphylococcus aureus* and *Staphylococcus hyicus/Staphylococcus agnetis*) are rarely isolated from ruminant mastitis (25), whereas CNS are often isolated and described as opportunistic pathogens (20), and *S. chromogenes* (found in this study) is one of the most commonly isolated CNS species in mastitis (25). CNS are part of the normal flora of the teat skin, and their role in bovine mastitis is not completely understood.

DNA sequencing used in this study also identified bacteria not yet acknowledged as mastitis pathogens, but present in this study at abundances, which warrant further investigation into their significance. In two study cows, *S. sanguinegens* was the most abundant bacterium in the mastitic quarter, exhibiting a significant increase in abundance in the absence of any known mastitis pathogen. Clinical infections caused by *S. sanguinegens* have rarely been previously reported, which may be to the fastidious nature of the organism (26) and its near-absence in culture-based studies (27, 28). *S. sanguinegens* has been found as part of the micro-flora of intra-amniotic infection in humans in which it was as prevalent as the most frequent invaders of the amniotic cavity (*Mycoplasma* spp.) (27), and using 16S rRNA gene sequencing, *S. sanguinegens* has also been identified in cases of septic arthritis (29) and late onset bronchiolitis obliterans syndrome (30). Thus, its pathogenic significance is becoming more appreciated. The classification of *S. sanguinegens* in the same family as *Fusobacteriaceae*, which contains known mastitis pathogens (31), further strengthens its possible classification as pathogenic.

Several bacterial genera are difficult to identify quickly by culture presenting circumstances in which genomic techniques could be advantageous. *Listeria* spp. have been previously identified in cases of mastitis, but conventional means of detection, while generally reliable, are expensive, laborious, and slow, requiring at least 3–7 days for a presumptive identification (32). *Listeria* spp. may even go undetected due to lack of suitable techniques employing specific media/antigens (33). *L. innocua* was detected and was significant in this study, and its zoonotic risk makes rapid and accurate identification crucial for reasons of public health and illustrates the value of rapid accurate identification by genomic techniques.

*Corynebacterium* spp. are among the most frequently isolated pathogens associated with subclinical mastitis in dairy cows (34), often being described as contagious. Specific species of *Corynebacterium* are sometimes difficult to identify in bacterial culture due to their slow-growing nature (35, 36). *Corynebacterium* spp. were identified here using DNA sequencing.

*Rhodococcus* species are rarely associated with mastitis in cattle, with only *Rhodococcus equi* being identified in a case of granulomatous mastitis (37). However, Watts et al. (38) demonstrated that *Rhodococcus* spp. were present in mastitic cases but had been misidentified as *Corynebacterium bovis* based on colony morphology. The sequencing techniques used in this study did identify *Rhodococcus* spp., but the changes in relative abundance were small.

*Enterococcus* spp. including *E. gallinarum* and *Enterococcus lactis* have been identified as causing/being associated with mastitis in several studies (4, 39). Routine bacteriological culture has been shown not to sufficiently discriminate all species of *Enterococcus* (36), yet differentiation is essential because of their antimicrobial resistance, with *E. gallinarum* being shown to have resistance to many commonly used antimicrobials (4). Conversely, in the case of *E. coli*, considered an opportunistic pathogen and associated with high daily milk yield and environmental exposure from bedding material, dirt, and management practices (20), several authors (40, 41) have reported that mild to moderate clinical mastitis cases caused by *E. coli* do not benefit from antimicrobial therapy.

Other bacteria were identified in the study at low abundances, demonstrating an increase in relative abundance between healthy and mastitic quarters and/or being of unknown significance with regard to mastitis. *M. lacumata* and *P. dagmatis* have not been identified as causing mastitis although it is known that *P. dagmatis* is a commensal organism found within the oral and gastrointestinal floras of many wild and domestic animals (42) and has been isolated in wounds originating from animal bites (43). *P. azotoformans*, found in one cow and exhibiting a relative abundance increase of 8.1, has not been identified as causative of bovine mastitis, but other *Pseudomonas* spp. such as *Pseudomonas aeruginosa*, have been (44).

Mastitic quarters in 12 cattle were not associated with a causative bacterium for which there are several possible explanations: some bacteria, e.g., *E. coli*, clear spontaneously (45) before testing and go undetected; mastitis can be caused by fungi and yeasts (46) or viruses, but 16S rRNA gene sequencing is limited only to the identification of bacteria. Additionally, if the genetic data are missing from the reference database for given bacteria they will be categorized as unclassified by 16S rRNA gene sequencing (47).

Admittedly, there are still some limitations to affordable metataxonomic sequencing. However, DNA sequencing technology has advanced at an incredible pace in recent years, leading to astonishing decreases in sequencing cost: at the scale of the whole human genome, the price per megabase has decreased by nearly an order of magnitude per year since 2001 (48). At such rates, it is not unlikely that in the very near future, metataxonomics will be a cost-effective diagnostic tool (8).

## Conclusion

Our metataxonomic approach enabled 80% of samples to be associated with a potential mastitis pathogen and identified lesser known pathogens, including at least one organism that may subsequently prove to be associated with mastitis in cattle (*S. sanguinegens*). The metataxonomic techniques are already not prohibitively costly and as the 16S rRNA genes databases continue to grow and sampling techniques improve, it is likely to become even less expensive and more attractive as a future technique in mastitis diagnostics.

## Author Contributions

JO analyzed data and wrote the manuscript draft. EG conducted the field study and the laboratory work and also critically revised the manuscript. SB assisted in data analysis and writing of the manuscript draft. RB conceived the study and critically revised the manuscript. GO corresponding author; conceived the study, assisted data analysis, and critically revised the manuscript. All authors approved the final version of the paper and agreed to be accountable for all aspects of the work.

## Conflict of Interest Statement

The authors declare that the research was conducted in the absence of any commercial or financial relationships that could be construed as a potential conflict of interest.

## Supplementary Material

Figure S1 **Mean relative abundance of the 25 most prevalent genera in samples diagnosed as *Corynebacterium* spp., *Staphylococcus carnosus, Escherichia coli*, and *Pastereulla dagmatis***.

Figure S2 **Mean relative abundance of the 25 most prevalent genera in samples diagnosed as *Moraxella lacumata, Faclamia hominis, Peptoniphilus methioninivorax*, and *Pseudomonas azotoformans***.

The research materials supporting this publication can be accessed by contacting the corresponding author.
