# Supplementary figures and images for: A Metataxonomic Approach Could Be Considered for Cattle Clinical Mastitis Diagnostics
Source: Front Vet Sci. 2017 Mar 10;4:36. doi: 10.3389/fvets.2017.00036 (PMC5344926; doi:10.3389/fvets.2017.00036)

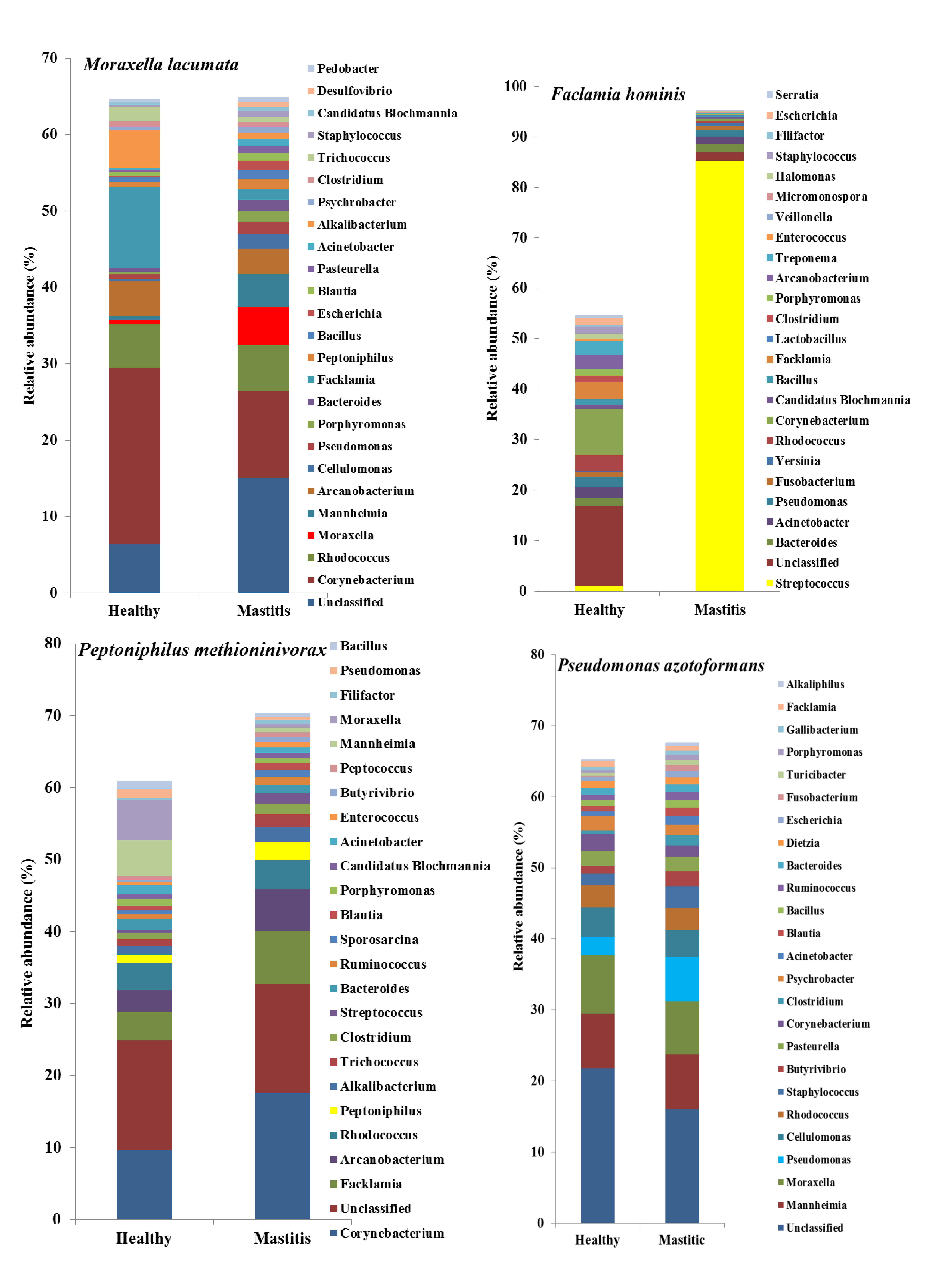

Supplement: Figure S2 — Mean relative abundance of the 25 most prevalent genera in samples diagnosed as Moraxella lacumata, Faclamia hominis, Peptoniphilus methioninivorax, and Pseudomonas azotoformans. [file Image_2.tif]
